# Human Body Fluid

**DOI:** 10.1155/2013/918793

**Published:** 2013-09-08

**Authors:** Shih-Bin Su, Terence Chuen Wai Poon, Visith Thongboonkerd

**Affiliations:** ^1^Department of Occupational Medicine, Chi Mei Medical Center, Tainan 710, Taiwan; ^2^Department of Paediatrics, Li Ka Shing Institute of Health Sciences, The Chinese University of Hong Kong, Prince of Wales Hospital, Shatin, New Territories, Hong Kong; ^3^Medical Proteomics Unit, Office for Research and Development, Faculty of Medicine Siriraj Hospital, and Center for Research in Complex Systems Science, Mahidol University, Bangkok, Thailand

Human body fluids are considered as attractive sources for clinical markers. As for disease diagnosis and prognosis, advantages of body fluid testing include low invasiveness, low cost, and rapid sample collection and processing. Besides, altered protein expression profiles in body fluids reflect the change of physiological states and cellular networks of the diseased tissue/organ. Thus, analysis of human body fluid has become one of the most promising approaches to discover biomarkers or reveal pathophysiological mechanisms for human diseases. Human body fluid analysis is inherently challenging due to their unique characteristics such as protein complexity and the wide dynamic range of protein abundances. With the remarkable advances in the methods for sample preparation, proteomics technology, and quantitation, it is now possible to analyze body fluids with higher sensitivity and robust experimental design. As the recognition of the importance of “Translational Medicine” which is the process of turning appropriate biological discoveries into drugs and medical devices that can be used in the diagnosis and/or treatment of patients, a great quantity of researches were focusing on the human sample including human body fluids. Thus, it is conceivable that the new insight unveiled by human body fluid analysis will attract a wide audience abroad including researchers from the field of basic sciences, bioinformatics, analytical chemistry, molecular biology, and hospital.

In the study of H. Y. Wu et al., authors performed the first differential proteomic profiling between peritoneal dialysates from chronic glomerulonephritis (CGN) patients at the early and middle stage of continuous ambulatory peritoneal dialysis (CAPD) treatment. The changed proteins provide clues to the PD-induced loss of proteins from the peritoneum and assist the identification of potential biomarkers for noninvasive monitoring of peritoneal damage.

M. H. Yang et al. also characterized the peritoneal dialysate proteins from diabetes mellitus (DM) by proteomic tools. CGN peritoneal dialysate was used as control. Differentially expressed proteins in DM samples may indicate a situation for possible drug treatment and predictors of peritonitis for a validation study in the future. 

P. Badiee reviewed the evaluation of different human body fluids, pleural Effusion, bronchoalveolar lavage fluid, peritoneal fluid, urine, pericardial effusion, blood, cerebrospinal fluid (CSF), synovial fluid, and saliva, for diagnosing fungal infections. Routine laboratory tests for the diagnosis of FI include urinalysis and blood analysis while analyses of both CSF and serum can improve the accuracy of the diagnosis.

K. Bořecká et al. studied the use of Coefficient of Energy Balance (CEB) values in a large CSF samples (*n* = 8183) to demonstrate that CEB enables more exact assessment of actual energy state in the CSF compartment than glucose and lactate alone. This study suggested that CEB combined with CSF cytology has a great importance for diagnosis, differential diagnosis, and early therapy of CNS diseases.

H. C. Yen et al. discussed potential interferences on the analysis of F_2_-IsoPs and F_4_-NPs in CSF by GC/NICI-MS by present analytical methods. Proper TLC purification for obtaining reliable chromatograms for F_2_-IsoPs quantification in CSF is suggested as well as the necessity of adding additional holding of the column at 280°C for a period of time following data acquisition during F_2_-IsoPs and F_4_-NPs analysis to avoid potential interferences on subsequent F_4_-NPs quantification in CSF.

